# The FERM and PDZ Domain-Containing Protein Tyrosine Phosphatases, PTPN4 and PTPN3, Are Both Dispensable for T Cell Receptor Signal Transduction

**DOI:** 10.1371/journal.pone.0004014

**Published:** 2008-12-24

**Authors:** Timothy J. Bauler, Wiljan J. A. J. Hendriks, Philip D. King

**Affiliations:** 1 Department of Microbiology and Immunology, University of Michigan Medical School, Ann Arbor, Michigan, United States of America; 2 Department of Cell Biology, Radboud University Nijmegen Medical Centre, Nijmegen, The Netherlands; Oklahoma Medical Research Foundation, United States of America

## Abstract

PTPN3 and PTPN4 are two closely-related non-receptor protein tyrosine phosphatases (PTP) that, in addition to a PTP domain, contain FERM (Band 4.1, Ezrin, Radixin, and Moesin) and PDZ (PSD-95, Dlg, ZO-1) domains. Both PTP have been implicated as negative-regulators of early signal transduction through the T cell antigen receptor (TCR), acting to dephosphorylate the TCRζ chain, a component of the TCR complex. Previously, we reported upon the production and characterization of PTPN3-deficient mice which show normal TCR signal transduction and T cell function. To address if the lack of a T cell phenotype in PTPN3-deficient mice can be explained by functional redundancy of PTPN3 with PTPN4, we generated PTPN4-deficient and PTPN4/PTPN3 double-deficient mice. As in PTPN3 mutants, T cell development and homeostasis and TCR-induced cytokine synthesis and proliferation were found to be normal in PTPN4-deficient and PTPN4/PTPN3 double-deficient mice. PTPN13 is another FERM and PDZ domain-containing non-receptor PTP that is distantly-related to PTPN3 and PTPN4 and which has been shown to function as a negative-regulator of T helper-1 (Th1) and Th2 differentiation. Therefore, to determine if PTPN13 might compensate for the loss of PTPN3 and PTPN4 in T cells, we generated mice that lack functional forms of all three PTP. T cells from triple-mutant mice developed normally and showed normal cytokine secretion and proliferative responses to TCR stimulation. Furthermore, T cell differentiation along the Th1, Th2 and Th17 lineages was largely unaffected in triple-mutants. We conclude that PTPN3 and PTPN4 are dispensable for TCR signal transduction.

## Introduction

A common event in cellular signal transduction is the phosphorylation of proteins on tyrosine residues which results in diverse cellular outcomes. This phosphorylation is mediated by protein tyrosine kinases (PTK). By contrast, protein tyrosine phosphatases (PTP) remove phosphate groups from protein tyrosyl residues and thus oppose the actions of PTK. The mammalian genome encodes 38 classical PTP that can be subdivided into receptor-like and non-transmembrane PTP [1]–[3]. The non-transmembrane PTP family consists of 17 members, of which 14 are expressed in T lymphocytes of the immune system [4].

T cells become activated subsequent to MHC-peptide recognition mediated by the clonally distributed, cell surface expressed T cell antigen receptor (TCR) [5]. One of the first events in the now well-established TCR signaling cascade is the phosphorylation and activation of the Src-family PTK, LCK and FYN [6]. These PTK phosphorylate immunoreceptor tyrosine-based activation motifs (ITAMs) present within the cytoplasmic tails of invariant CD3 and TCRζ proteins that form part of the TCR complex [7]. Subsequently, the Syk-family kinase, ZAP-70, is recruited to the complex by the recognition of phosphorylated ITAMs, and, in turn, is activated via Src-family PTK-mediated phosphorylation. Activated ZAP-70 phosphorylates the transmembrane adapter protein, linker for activation of T cells (LAT) [8]. LAT further propagates the signal, leading to membrane recruitment of additional signaling intermediates that ultimately result in the nuclear mobilization of the transcription factors NFAT, NF-κB, and AP-1 [9]. These transcription factors drive the expression of new genes that result in cytokine secretion, cytokine receptor expression, cell division, and effector cell differentiation.

While the role of PTK in TCR signal transduction has been extensively studied, the identity of PTP that negatively-regulate this pathway is less clear. PTP that are established physiological negative-regulators of proximal TCR signaling are SHP-1 and PEP. These PTP dephosphorylate and inactivate LCK, FYN, and ZAP-70 [10]–[12]. Other PTP that have been implicated in negative regulation of TCR signal transduction are PTPN3 and PTPN4 [13], [14]. In mice, these PTP are 50% identical and 67% homologous at the amino acid level. They consist of an NH_2_-terminal FERM (Band 4.1, Ezrin, Radixin, and Moesin) domain, a central PDZ (PSD-95, Dlg, ZO-1) domain, and a COOH-terminal PTP domain. FERM and PDZ domains bind the cytosolic domain of transmembrane proteins [15]–[17]. Both domains have also been shown to bind directly to the phospholipid phosphatidylinositol 4,5 biphosphate (PIP_2_) [18], [19]. The FERM domains of PTPN3 and PTPN4 are required for PTP membrane localization in T cells [20]. A screen in the Jurkat T cell leukemia line seeking to identify candidate negative regulators of TCR signal transduction revealed that over-expression of PTPN3 and PTPN4 resulted in an approximate 75% and 40% reduction, respectively, of TCR-induced activation of the promoter for the T cell growth-promoting cytokine, IL-2 [21]. Mutation of the catalytic cysteine residue or deletion of the FERM domain from these PTP abrogated this inhibitory effect, illustrating the importance of these domains for negative regulation [20], [21]. In a separate study, PTPN3 was shown to both bind and dephosphorylate TCRζ *in vitro* and when over-expressed in COS fibroblasts [22]. Recently, PTPN4 has also been shown to dephosphorylate TCRζ [23].

The third member of the FERM and PDZ domain-containing PTP family is PTPN13, also known as PTP-Bas, PTP-BL, and FAP-1 [24]. PTPN13 is a large protein that in addition to a PTP domain contains one FERM domain, five PDZ domains and a non-catalytic C-lobe domain. Mice that express a PTP domain-deleted form of PTPN13 exhibit impaired motor nerve repair and axon branching, and retinal ganglion cell neurite initiation and survival [25], [26]. This suggests a role for PTPN13 in neural cell growth and survival, especially following injury. Additionally, *in vitro*, T cells from PTPN13-deficient mice demonstrated increased differentiation into T helper-1 (Th1) and T helper-2 (Th2) subsets that synthesize the cytokines interferon-γ (IFN-γ) and IL-4 respectively [27]. Ostensibly, this is associated with increased phosphorylation of signal transducer and activator of transcription (STAT) proteins which mediate cytokine signal transduction.

We have previously shown that mice lacking PTPN3 exhibit normal T cell development and function [28]. We hypothesized that the lack of apparent function of PTPN3 in T cells can be explained on the grounds that PTPN4 can substitute for the function of PTPN3 in this cell type. To address this we have generated PTPN4-deficient mice and PTPN4/PTPN3 double-deficient mice. Furthermore, to address the possibility that PTPN13 can substitute for the function of both PTPN4 and PTPN3 in T cells, we have additionally generated PTPN4/PTPN3/PTPN13 triple-mutant mice. The function of T cells in these different mice has been examined.

## Materials and Methods

### Mice

The embryonic stem (ES) cell line, OST146128 (of 129 Sv/Ev origin), which contains a gene trap cassette within intron 2 of the *ptpn4* locus, was purchased from Lexicon Genetics (The Woodlands, TX). ES cells were injected into C57BL/6J×(C57BL/6J×DBA/2) blastocysts to generate chimeras, which were bred to C57BL/6 mice to achieve germline transmission of the trapped *ptpn4* allele. F1 progeny were then intercrossed to generate PTPN4-deficient mice for experiments. To generate PTPN4/PTPN3 double-deficient mice, PTPN4 F1 mice were crossed to *ptpn3 ^tm1PdK/tm1PdK^* mice (C57BL/6 background) described previously [28]. Resulting PTPN4/PTPN3 double heterozygotes were then intercrossed to generate double-deficient animals for experiments, or were crossed to *ptpn13 ^ΔPTP/ΔPTP^* (C57BL/6 background) mice also described previously [25]. In the latter case, progeny were intercrossed twice to concentrate the targeted alleles, and then intercrossed again to generate PTPN4/PTPN3-deficient PTPN13 ΔPTP/ΔPTP triple-mutant mice. Unless otherwise noted, all mice were 6–8 weeks of age at the time of experimentation. All experiments were performed in compliance with University of Michigan guidelines and were approved by the University Committee on the Use and Care of Animals.

### PCR

To confirm loss of PTPN4 expression in PTPN4 gene-trapped mice, RNA was purified from splenocytes using Trizol (Invitrogen) and analyzed by RT-PCR (Superscript One-Step kit; Invitrogen) using a forward primer based in exon 2 and a reverse primer based in exon 8 of the *ptpn4* gene. βActin transcripts were also amplified to control for the quantity and quality of RNA preparations.

Relative expression levels of PTPN4 in different tissues of wild-type mice were determined by quantitative PCR of cDNA samples from a Tissue Scan RT kit (Origene). Amounts of cDNA used in reactions were normalized to expression levels of GAPDH in each tissue. SYBR Green mastermix (Eurogentec) was used with a forward primer based in exon 2 and a reverse primer based in exon 3 of the *ptpn4* gene and reactions were performed in an MX3000p quantitative real-time PCR machine (Stratagene). Cycle threshold values were used to calculate the relative abundance of PTPN4 transcripts in tissues. Results were normalized to the amount of PTPN4 transcript detected in muscle, which was arbitrarily given a value of one.

### Flow cytometry

Thymocytes, splenocytes, and lymph node (LN) cells were blocked with murine IgG (Sigma) and stained with the following conjugated monoclonal antibodies (BD Biosciences): H57-597-APC (TCRβ chain), RA3-6B2-APC-Cy7 (CD45R/B220), IM7-FITC (CD44), GK1.5-APC-Cy7 (CD4), 53-6.7-PerCP (CD8) and PC61-PE (CD25). Cell staining was analyzed by flow cytometry using a FACSCanto (Becton Dickinson).

### T cell cytokine synthesis and proliferation

LN cells were stimulated with varying concentrations of 145-2C11 (CD3ε; eBioscience) and 37.51 (CD28; BD Biosciences) in 96 well round-bottom plates in complete medium (RPMI supplemented with 10% FCS, 25 nM β-mercaptoethanol, 50 U/mL Penicillin, 50 mg/mL Streptomycin, 2 mM L-glutamine, 10 mM HEPES, and 1 mM sodium pyruvate). Concentrations of cytokines in supernatants were determined by ELISA after 24 h (IL-2) or 48 h (IFN-γ and IL-4) of culture. To assess T cell proliferation, splenocyte cultures were first labeled with 1 µM carboxyfluorescein succinimidyl ester (CFSE; Molecular Probes), and stimulated as above. After 72 h stimulation, CFSE fluorescence intensity was analyzed by flow cytometry.

In some experiments T cells were induced to differentiate into Th1, Th2, or Th17 cells. For this purpose, splenic CD4+ T cells were purified by negative selection and were stimulated with immobilized anti-CD3 (3 µg/mL for coating) and soluble (1 µg/mL) anti-CD28 antibodies plus recombinant human IL-2 (50 U/mL) in complete medium. For Th1 and Th2 and differentiation, IL-12 (3.5 ng/mL)/anti-IL-4 antibody (11B11; 10 µg/mL) or IL-4 (10 ng/mL)/anti-IFN-γ antibody (R4-6A2; 10 µg/mL), respectively, were added to the cultures. To induce Th17 cell differentiation, anti-IFN-γ (10 µg/mL), anti-IL-4 (10 µg/mL), IL-23 (20 ng/mL), IL-6 (10 ng/mL), and TGFβ (5 ng/mL) were added to media. After 72 h, cells were recultured in complete medium supplemented with IL-2 for Th1 and Th2 polarized cultures or anti-IL-4/anti-IFN-γ/IL-23/IL-6 for Th17 polarized cultures as above. After a further 48 h, cells were restimulated with immobilized CD3 antibody (1 µg/mL for coating), and supernatants were harvested after 24 h for quantitation of cytokine secretion by ELISA. All cytokines and blocking antibodies were purchased from PeproTech and BD Biosciences, respectively.

### Statistical analysis

Statistical significance of differences in cell population representation or in cytokine secretion between test and control mice was assessed using a paired or unpaired Student's T-test as indicated.

In T cell polarization studies, in order to compile data from a number of independent experiments, cytokine secretion by different PTP mutant T cells was first normalized to that observed with control T cells to derive a fold increase in cytokine secretion as follows: (PTP mutant secretion–control secretion)/control secretion (in the case of increased secretion by mutant; assigned a positive value) or (control secretion–PTP mutant secretion)/PTP mutant secretion (in the case of decreased secretion by mutant; assigned a negative value). Mean fold increase in cytokine secretion relative to wild-type secretion was then calculated for several repeat experiments. Statistical significance of differences in cytokine secretion between PTP mutant and control cells was determined with the use of a paired Students T-test.

### Western blotting

Splenocytes were lysed in a buffer containing 1% NP-40, run on SDS-PAGE gels and transferred to PVDF membranes (Perkin Elmer). Membranes were then probed with an anti-PTPN3 mAb [28], before stripping and reprobing with a GAPDH antibody (FL-335; Santa Cruz) to determine the amount of total protein loaded in each lane.

## Results

### Generation of PTPN4-deficient mice

**Figure 1:**
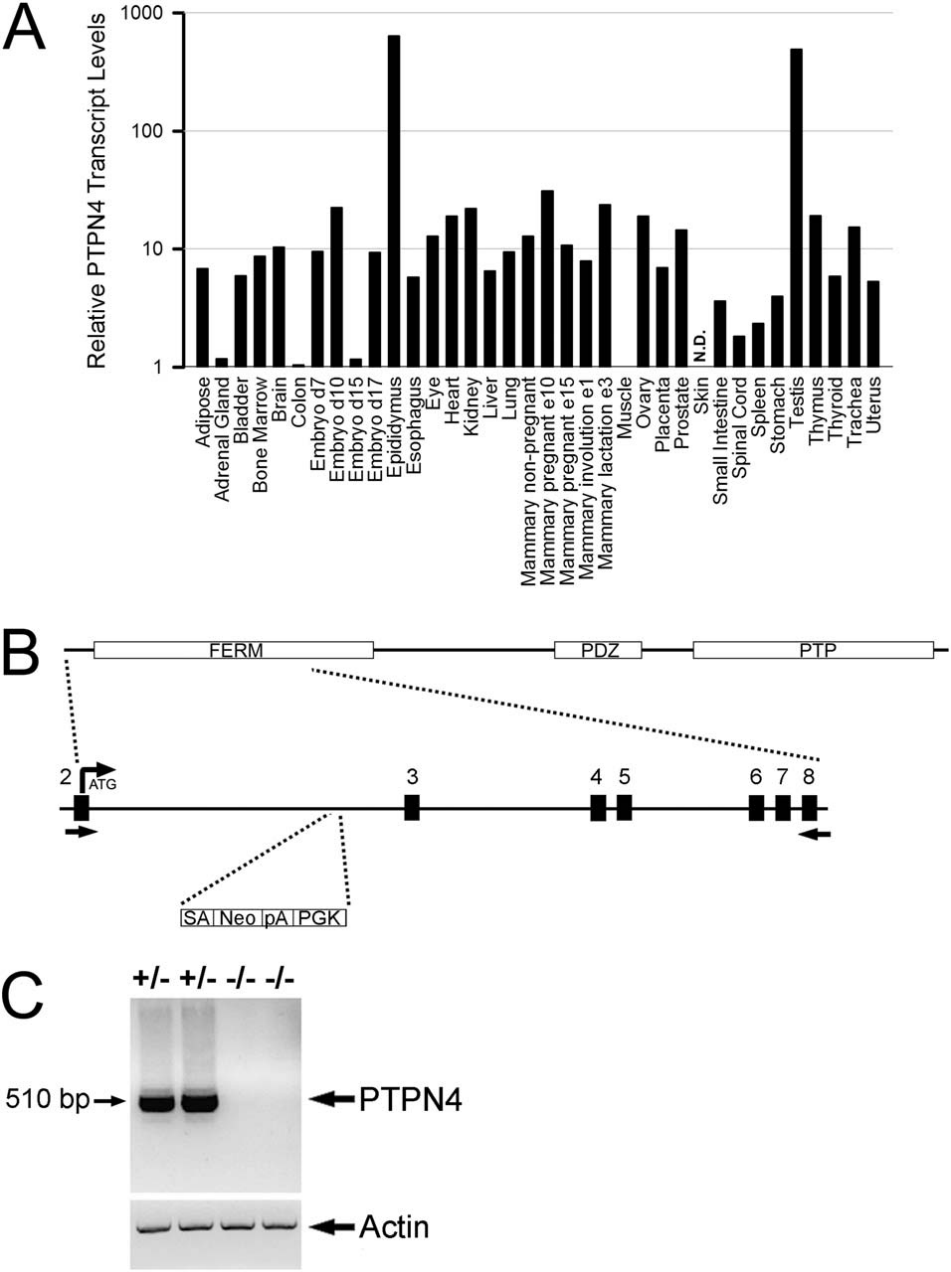
Generation of PTPN4-deficient mice. A) Relative PTPN4 expression levels were determined by quantitative PCR of different tissue cDNA samples using PTPN4-specific primers. For each tissue, expression levels are normalized to muscle, arbitrarily given a value of 1. N.D., not detected. B) Top, Domain organization of PTPN4, showing the location of the FERM, PDZ, and PTP domains. Bottom, Exon/intron organization of the 5′ end of the ptpn4 gene encoding part of the PTPN4 FERM domain. The location of the gene-trapping cassette within intron 2 is shown. Note that exon 2 contains the translation initiation site. Arrows indicate positions of PCR primers used in panel C. SA, splice acceptor; Neo, sequence encoding neomycin phosphotransferase; pA, polyadenylation sequence; PGK, cloning vector component of gene trap. C) RTPCR was performed upon RNA isolated from splenocytes from mice heterozygous and homozygous for the gene-trapped allele using PTPN4 primers shown in B or control β-actin-primers. Note the complete absence of PTPN4 transcripts in the homozygous mice.

PTPN4 is a member of the non-transmembrane PTP family that contains both a FERM and PDZ domain. A previous report had indicated that PTPN4 is strongly expressed in testis [29]. To analyze which other tissues also expressed PTPN4, we performed quantitative PCR upon a panel of normalized mouse tissue cDNA samples using PTPN4-specific primers (Figure 1A). As shown, PTPN4 transcript levels were indeed highest in male reproductive organs. In addition, however, PTPN4 was also expressed in most other examined tissues including thymus and spleen.

To study the role of PTPN4 in T cells, we generated PTPN4-deficient mice. For this purpose, we obtained an ES cell line that contained a gene trap inserted within the *ptpn4* locus. This ES cell line was used to generate chimeric founder mice which were crossed to wild-type mice to achieve germline transmission of the trapped allele. Heterozygote PTPN4 mice were then intercrossed to generate homozygous PTPN4 mutants. Homozygote mutants were born in the expected Mendelian ratios and displayed no abnormalities in growth or development. Both male and female mice were fertile.

The PTPN4 gene trap is contained within intron 2 of the *ptpn4* locus, downstream of exon 2 that contains the translation initiation codon (Figure 1B). Consequently, in primary transcripts produced from the gene-trapped allele, exon 2 would be spliced to the gene trap which contains a strong splice acceptor at its 5′end. This would result in extinction of PTPN4 protein expression since read through of exon 2 to exon 3 would not occur. To confirm loss of PTPN4 expression, we performed RTPCR upon splenic RNA from heterozygote and homozygote gene-trapped mice using a forward primer based in exon 2 and a reverse primer based in exon 8 of the *ptpn4* gene (Figure 1C). As shown, PTPN4 amplicons of the expected size were readily detected in heterozygote mice but were completely absent from homozygote mice. Thus, homozygous PTPN4 gene-trapped mice are PTPN4 null and cannot express PTPN4 protein.

### Intact immune compartments in PTPN4-deficient mice

**Figure 2:**
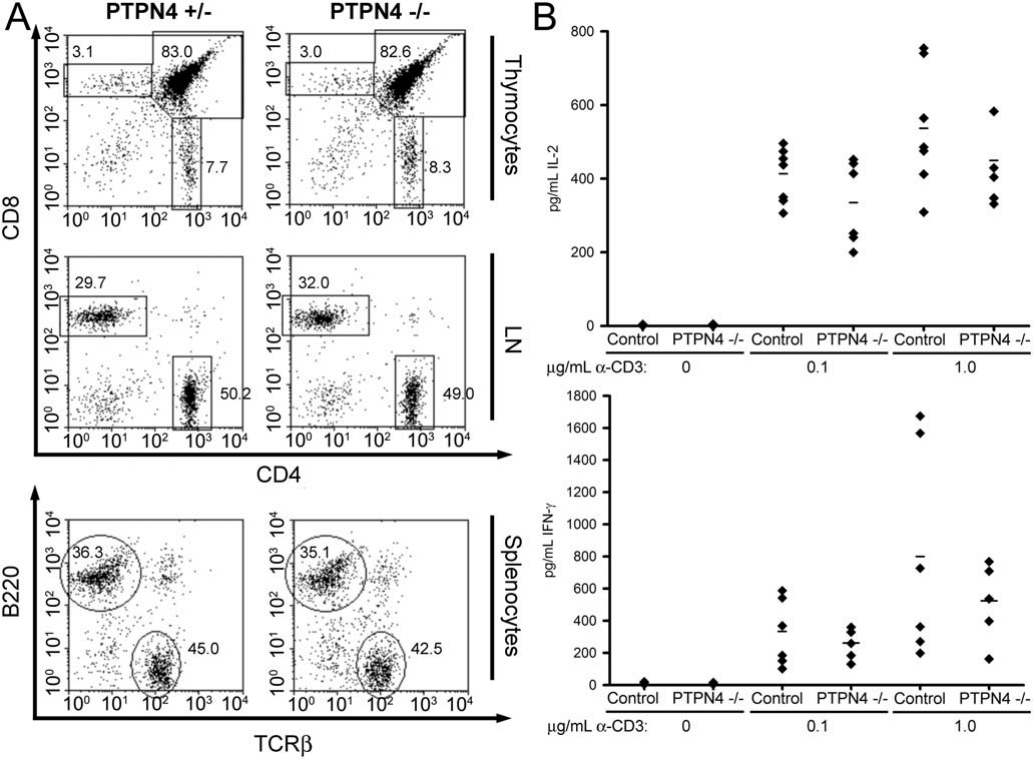
Normal T cell development and function in PTPN4-deficient mice. A) Flow cytometry plots of thymocytes, LN cells, and splenocytes from PTPN4-deficient mice and littermate controls showing expression of the indicated markers on live cell populations. Percentages of cells that fall within the indicated regions are shown. Data are representative of three repeat experiments. B) LN T cells were stimulated with the indicated concentrations of CD3 antibody and 0.5 µg/mL CD28 antibody. Concentrations of cytokines in supernatants were determined by ELISA. Each symbol represents the mean of triplicate determinations from a single mouse. Bars represent the mean cytokine secretion. Differences between PTPN4-deficient and control mice are not statistically significant (Paired Student's T test).

In order to investigate a potential role for PTPN4 in immune cell development, we examined the numbers and ratios of leukocytes in primary and secondary lymphoid organs of PTPN4-deficient mice by flow cytometry (Figure 2A). With regards T cell development in the thymus, no differences were observed in the number or ratio of CD4−CD8− double-negative (DN), CD4+CD8+ double-positive (DP) or CD4+CD8− or CD4−CD8+ single-positive (SP) thymocytes compared to controls. Likewise, no differences in the number or ratio of CD4+ or CD8+ T cells were observed in LN or spleen. All other examined leukocytic populations including B cells, macrophages, dendritic cells, granulocytes and NK cells were also normally represented in secondary lymphoid organs as well as bone marrow of PTPN4-deficient mice (Figure 2A and not shown).

### T cell activation and function in PTPN4-deficient mice

We next examined the ability of PTPN4-deficient T cells to synthesize cytokines in response to TCR stimulation. LN cells from PTPN4-deficient and control mice were thus stimulated with CD3 antibodies (directed to the TCR complex) and antibodies against the CD28 T cell costimulatory receptor. Secretion of cytokines into culture supernatants was then measured by ELISA. No significant differences in the synthesis of the T cell growth factor, IL-2, or the hallmark Th1 and Th2 cytokines, IFN-γ and IL-4, respectively, were apparent in these experiments (Figure 2B and data not shown). Thus, PTPN4 appears to be dispensable for TCR-induced synthesis of these cytokines.

### T cell development and function in PTPN4/PTPN3 double-deficient mice

**Figure 3:**
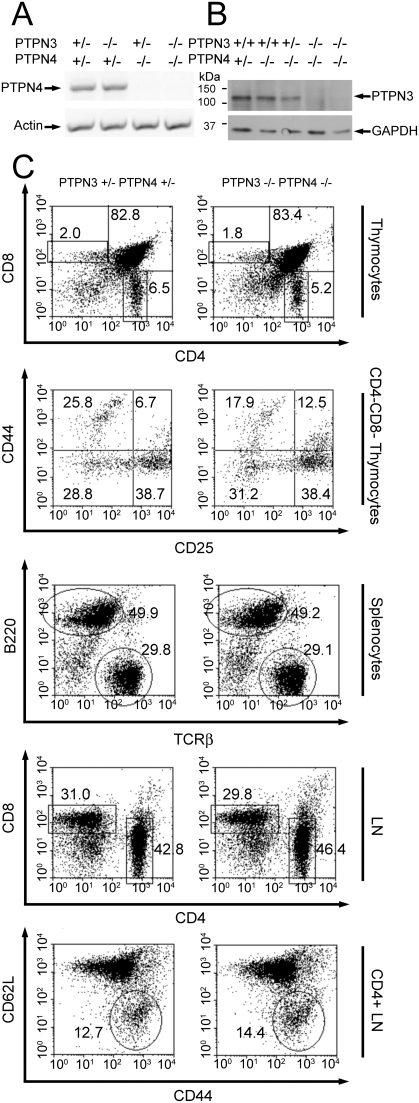
PTPN4/PTPN3 double-deficient mice show normal T cell development. A) Expression of PTPN4 in mice of the indicated genotypes (top) determined by RTPCR. B) Expression of PTPN3 in mice of the indicated genotypes (top) determined by Western blotting. Blots were stripped and reprobed with GAPDH antibodies to confirm equivalent protein loading. C) Flow cytometry plots of thymocytes and peripheral immune cells from PTPN4−/−PTPN3−/−mice and littermate controls showing expression of the indicated markers on live cell populations. In CD44/CD62L plots, the gated population represents recently activated memory T cells. Data are representative of six repeat experiments.

We hypothesized that the apparent lack of a T cell phenotype in PTPN4-deficient mice can be explained by a functional redundancy of PTPN4 with PTPN3 and vice versa. Analysis of the expression of PTPN3 in PTPN4-deficient mice and of PTPN4 in PTPN3-deficient mice indicated that loss of expression of one PTP did not result in a compensatory increase in expression of the other PTP (Figure 3 A and B). Nonetheless, the possibility of functional redundancy still existed. Therefore, PTPN4-deficient mice were crossed with PTPN3-deficient mice and progeny were then intercrossed to generate PTPN4/PTPN3 double-deficient mutants.

Like single PTP-deficient mice, PTPN4/PTPN3-double-deficient mice were born in expected Mendelian ratios and showed normal growth and development. With regards T cell development, no abnormalities were apparent (Figure 3C). Numbers and ratios of DN, DP and SP thymocyte subpopulations were normal and among DN cells, the representation of CD44+CD25− (DN1), CD44+CD25+ (DN2), CD44loCD25+ (DN3) and CD44−CD25− (DN4) subsets was unaffected in the double-mutants. Similarly, in secondary lymphoid organs, numbers and ratios of CD4+ and CD8+ T cells were normal. In addition, there was no evidence of any increased previous or ongoing T cell activation as judged by an increase in the frequency of cells that express the CD44 memory cell marker or a decrease in the frequency of cells that express CD62L, respectively.

**Figure 4:**
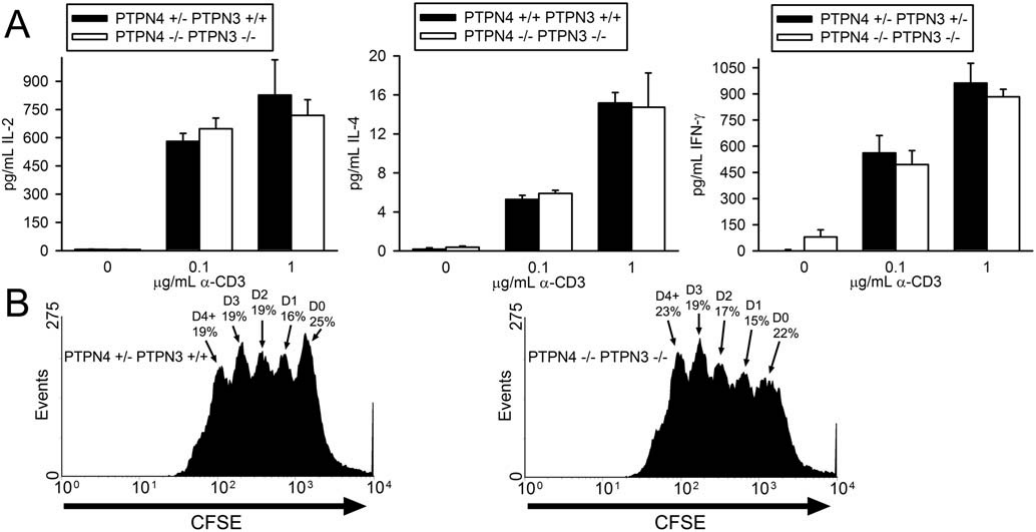
T cell cytokine synthesis and proliferation in PTPN4/PTPN3 double-deficient mice. A) LN T cells were stimulated with the indicated concentrations of CD3 antibody and 0.75 µg/mL CD28 antibody. Concentrations of cytokines in supernatants were determined as in Figure 2. Each bar represents the mean plus one standard deviation of triplicate determinations from one mouse. Differences between mice are not statistically significant (Student's T-test). Data are representative of six repeat experiments. B) Splenic T cells were labeled with CFSE and stimulated with 0.1 µg/mL CD3 antibody and 0.75 µg/mL CD28 antibody. After 72 h, CFSE dye intensity was measured by flow cytometry. The percentage of live cells that have undergone the indicated number of cell divisions is shown. Data are representative of three repeat experiments.

To examine the influence of loss of expression of PTPN4 and PTPN3 upon T cell function, we examined both T cell cytokine synthesis and T cell proliferation in PTPN4/PTPN3 double-deficient mice. Synthesis of IL-2, IFN-γ and IL-4 in response to CD3/CD28 antibody stimulation was determined as before (Figure 4A). As shown, T cells from PTPN4/PTPN3 double-deficient mice secreted similar quantities of these cytokines in these assays as T cells from control mice. To assess division, T cells were labeled with CFSE and dilution of CFSE was determined 72 h post-CD3/CD28 stimulation (Figure 4B). These analyses revealed that PTPN4/PTPN3 double-deficient T cells proliferate comparably to control T cells *in vitro*.

### Generation and characterization of PTPN4/PTPN3 double-deficient PTPN13 ΔPTP/ΔPTP mice

**Figure 5:**
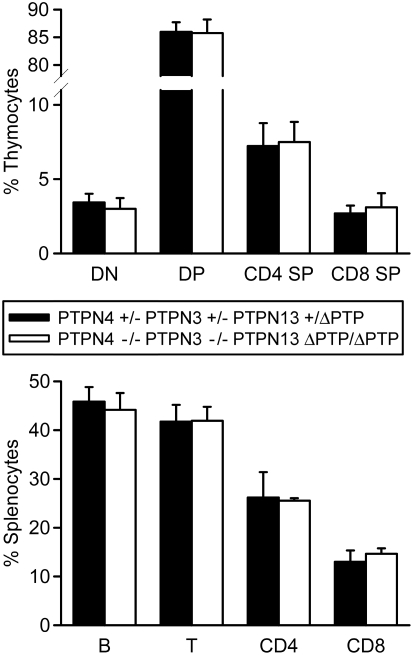
Normal T cell development in PTPN4/PTPN3 double-deficient PTPN13 ΔPTP/ΔPTP mice. Thymocytes and splenocytes from mice of the indicated genotypes were analyzed by flow cytometry for expression of T cell and B cell markers. Shown is the mean percentage representation plus one standard deviation of the indicated subpopulations among total live cells (n = 4 mice of each genotype). Differences between mice are not statistically significant.

**Figure 6:**
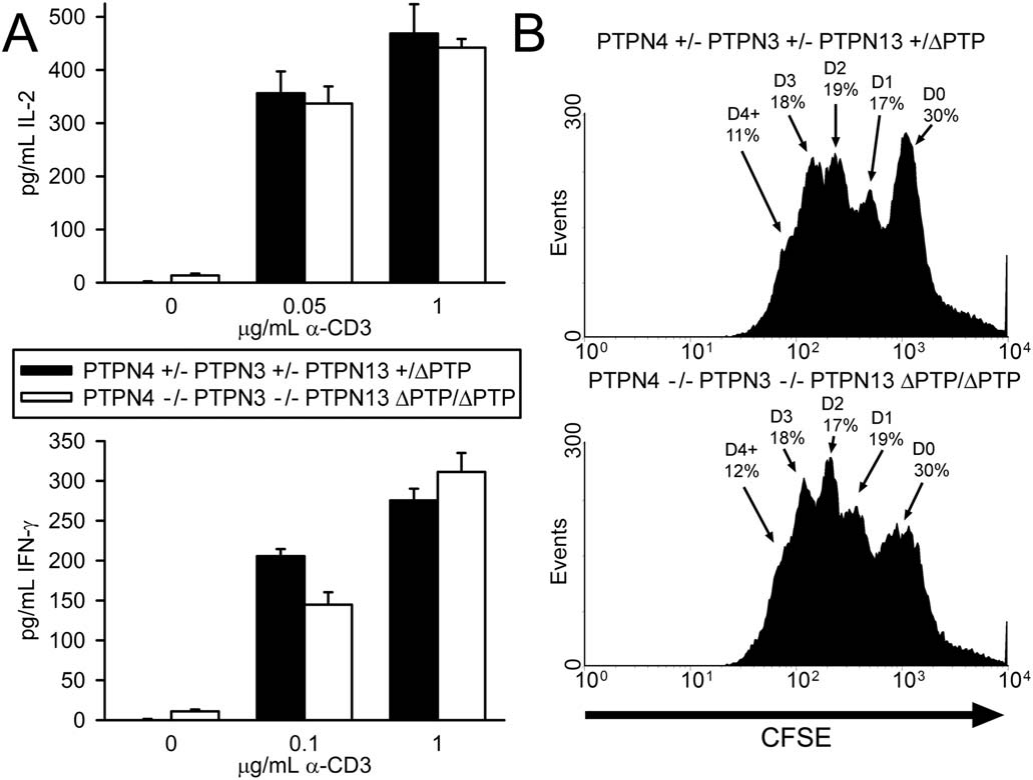
Function of PTPN4/PTPN3 double-deficient PTPN13 ΔPTP/ΔPTP T cells. A) LN T cells were stimulated with the indicated concentrations of CD3 antibody and 0.5 µg/mL CD28 antibody. Concentrations of cytokines in supernatants were determined as in Figure 2. Differences between mice are not statistically significant, excepting IFN-γ secretion at low dose anti-CD3 which is not a reproducible finding over four repeat experiments. B) Splenic T cells were labeled with CFSE and stimulated with 0.1 µg/mL CD3 antibody and 0.5 µg/mL CD28 antibody. After 72 h, CFSE dye intensity was measured by flow cytometry. Data are representative of four mice of each genotype.

The remaining PTP in the mouse and human genomes that contains both FERM and PDZ domains is PTPN13. Although the degree of homology between PTPN13 and PTPN4 or PTPN3 is much less than that between PTPN4 and PTPN3, the possibility that PTPN13 might compensate functionally for the loss of PTPN4 and PTPN3 in T cells existed. Moreover, recent studies of PTPN13-deficient mice indicated that this PTP regulates T helper cell differentiation through function as a negative-regulator of the phosphorylation of STAT-4 and STAT-6. Therefore, we asked if a role for PTPN4 and PTPN3 in T cells might be revealed in mice that do not express functional PTPN13. To examine this, PTPN4/PTPN3 double-deficient mice were crossed with mice that express a PTP domain-deleted PTPN13 protein (PTPN13 ΔPTP) to generate PTPN4/PTPN3-double-deficient PTPN13 ΔPTP/ΔPTP mutant mice [25]. As with single PTP and PTP double-deficient mutants, PTPN4/PTPN3 double-deficient PTPN13 ΔPTP/ΔPTP mice were born in normal Mendelian ratios and showed normal growth and development. With regards T cell development, analysis of the numbers and ratios of T cell subsets in thymus and peripheral lymphoid organs showed that this was also normal in triple-mutant mice (Figure 5). In addition, peripheral T cells from triple-mutants were found to secrete similar amounts of cytokines and proliferated to the same extent in response to CD3/CD28-stimulation as T cells from control mice (Figure 6).

**Figure 7:**
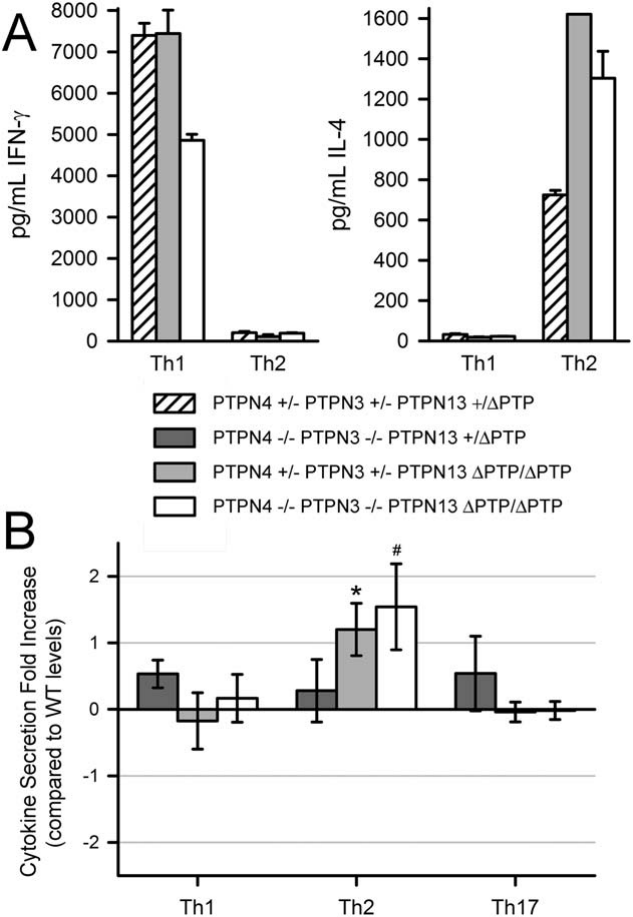
PTPN4 and PTPN3 are not required for CD4+ T cell differentiation. Purified CD4+ T cells were cultured under Th1-, Th2-, or Th17-inducing conditions. T cells were re-stimulated with CD3 antibodies and concentrations of IFN-γ, IL-4, and IL-17, respectively, in culture supernatants was determined by ELISA. A) Shown are results of a representative Th1/Th2 polarization experiment comparing control, PTPN13 ΔPTP/ΔPTP and PTPN4/PTPN3 double-deficient PTPN13 ΔPTP/ΔPTP T cells. Shown is mean cytokine secretion plus 1 standard deviation of triplicate determinations from single littermate mice. Conditions of polarization are indicated on the x-axis. B) Several repeat experiments of the type indicated in A) were performed. In each experiment, a fold increase in IFN-γ, IL-4 or IL-17 expression under conditions of Th1, Th2 or Th17 polarization respectively was calculated for mutant T cells relative to wild-type T cells (see Materials and Methods). Shown is the mean fold increase+/−one standard error (PTPN4−/−PTPN3−/−PTPN13+/ΔPTP, n = 8; PTPN4+/−PTPN3+/−PTPN13 ΔPTP/ΔPTP, n = 5; PTPN4−/−PTPN3−/−PTPN13 ΔPTP/ΔPTP, n = 4). note that a 0 value fold increase indicates no change in cytokine secretion relative to wild-type. * differences relative to wild-type responses are statistically significant as determined in a paired Student's T-test comparing raw data (see Materials and Methods). All other differences relative to wild-type cells are not statistically significant. # p<0.07.

Given the reported role of PTPN13 in control of T helper cell differentiation, we examined this directly in the triple-mutants. T cells from wild-type, PTPN13 ΔPTP/ΔPTP, PTPN4/PTPN3 double-deficient, and PTPN4/PTPN3 double-deficient PTPN13 ΔPTP/ΔPTP mice were thus induced to differentiate along Th1, Th2 or Th17 lineages (see Materials and Methods). To examine the extent of differentiation, cells were then re-stimulated with CD3 antibody and secretion of IFN-γ, IL-4 and IL-17, respectively, was determined by ELISA (Figure 7). Results of these analyses showed that T cells from PTPN3/PTPN4-double-deficient mice were able to differentiate along the Th1, Th2 and Th17 lineages to an extent comparable to that observed with T cells from wild-type mice. Similarly, T cells from PTPN13 ΔPTP/ΔPTP mice showed the same extent of differentiation into Th1 and Th17 cells as T cells from wild-type mice, although showed increased differentiation along the Th2 cell lineage. These last results, therefore, differ from previous findings where increased Th1 differentiation as well as increased Th2 differentiation was reported in PTPN13-deficient mice. Finally, and importantly, T cells from PTPN4/PTPN3 double-deficient PTPN13 ΔPTP/ΔPTP mice behaved similarly to T cells from PTPN13 ΔPTP/ΔPTP mice in so much that they showed normal differentiation into Th1 and Th17 cells and somewhat increased differentiation along the Th2 lineage (albeit not reaching statistical significance, p<0.07). In summary, these results show that PTPN3 and PTPN4 are dispensable for T cell function, even in the absence of functional PTPN13.

## Discussion

The non-receptor PTP, SHP-1 and PEP, are established physiological negative-regulators of TCR signal transduction that act by dephosphorylating PTK that become activated at an early point in the TCR signaling cascade [10]–[12]. However, the identity of PTP which dephosphorylate other tyrosine-phosphorylated proteins in this cascade, and which may thus also function as negative-regulators of TCR signaling, has remained elusive. In this regard, there has been considerable interest in two closely related PTP, PTPN3 and PTPN4, as potential novel negative-regulators of TCR signal transduction. Both PTP are able to dephosphorylate the TCRζ chain *in vitro* and in cell lines [22], [23]. In addition, when over-expressed in the Jurkat T leukemia cell line, both PTP inhibit TCR-induced activation of the IL-2 promoter, an effect that requires an intact FERM domain necessary for translocation of these PTP to the plasma membrane [20], [21].

To address if PTPN3 functions as a physiological negative-regulator of TCR signal transduction, we recently generated PTPN3-deficient mice [28]. However, no abnormalities in T cell development or function were apparent in these mice. We hypothesized that the lack of a T cell phenotype could be explained by a functional redundancy of PTPN3 with PTPN4, since both PTP are expressed in T cells. Hence, in the current study, we generated PTPN4-deficient and PTPN3/PTPN4 double-deficient mice. Consistent with a recent report, T cell development and function was found to be normal in PTPN4-deficient mice [23]. Moreover, there was no apparent T cell phenotype in PTPN3/PTPN4 double-deficient mice either. Thus, PTPN4 does not compensate for the loss of PTPN3 in T cells and vice versa.

We further asked if the absence of a T cell phenotype in PTPN3/PTPN4 double-deficient mice could be explained by functional redundancy with PTPN13, which also contains FERM and PDZ domains, albeit that this PTP shows a low degree of homology to PTPN3 and PTPN4. Therefore, we generated PTPN4/PTPN3 double-deficient PTPN13 ΔPTP/ΔPTP mice that lack functional forms of all three PTP. Once again, T cell development and TCR-induced cytokine secretion and proliferation were found to be normal in these mice. In addition, since PTPN13 has previously been shown to inhibit Th1 and Th2 differentiation through dephosphorylation of STAT proteins, we examined T helper cell polarization in triple-mutants [27]. In comparison to T cells from control mice, T cells from triple-mutant mice showed normal differentiation into Th1 and Th17 cells. An increased (not statistically significant) propensity of T cells to differentiate into Th2 cells was observed in triple-mutants. However, this increased Th2 differentiation was no greater in magnitude than that observed with PTPN13 ΔPTP/ΔPTP T cells. Thus, in conclusion, we have found no evidence for the notion that PTPN3 and PTPN4 function as negative-regulators of TCR signal transduction.

Functions for PTPN3 and PTPN4 outside of the immune system have been proposed. PTPN3 has been shown to dephosphorylate the growth hormone receptor (GHR) and one other group reported an increased weight gain amongst male PTPN3-deficient mice [30], [31]. These findings suggest a role for PTPN3 as a negative-regulator of GHR signal transduction and body mass. However, counter to this, in our colony, we have not observed any increased growth of male or female PTPN3-deficient mice up to 18 months of age. Similarly, we have not observed any increased body mass of PTPN4-deficient or PTPN4/PTPN3 double-deficient mice in comparison with littermate controls (not shown). It is possible that this discrepancy can be explained in terms of differences in genetic background or the precise nature of genetic disruptions in PTPN3 mutant strains. With regards strain differences, PTPN3 mutant mice that we have studied include PTPN3 gene-trapped mice on a 129P2/Ola Hsd background and PTPN3 gene-targeted mice on a C57BL/6 background (neither show growth abnormalities) [28]. By contrast, in the study in which an increased weight gain was reported for PTPN3-deficient males, mice were on a mixed 129 SvEv×C57BL/6 genetic background [31]. Concerning differences in the precise nature of disruptions, of the two PTPN3 mutant mice that were generated in our laboratory, we established by Western blotting that expression of PTPN3 protein was extinguished. By contrast, in the third PTPN3 mutant strain, although the nature of the genetic mutation precludes expression of catalytically-active PTPN3 protein, whether or not these mice express truncated forms of PTPN3 that contain only modular binding domains remains to be established.

Aside from growth, PTPN3 and PTPN4 have both been ascribed functions within the central nervous system. PTPN4 interacts with glutamate receptor subunits, which are required for neural development and function [32]. Consistent with a neural role, PTPN4-deficient mice were shown to have impaired cerebellar function, including defective motor learning [33]. In a recent report, PTPN3-deficient mice were also shown to have impaired memory function and motor learning, albeit that significant differences compared to controls were detected only in females [34]. Furthermore, in *Drosophila*, loss of expression of PTPMEG (the single homologue of PTPN3 and PTPN4 in this species) results in defective axon growth associated with impaired development of mushroom bodies [35]. Therefore, given these reports, we examined motor learning function in our own PTPN4 and PTPN3 single-deficient mice and in PTPN4/PTPN3 double-deficient mice. In an accelerated rotarod test, no difference in motor learning was noted in comparison with controls (not shown). For PTPN3, discrepancies might again be explained by differences in background strain or the precise nature of genetic disruptions. For PTPN4, only strain differences could be invoked to account for the discrepant findings. Thus, the PTPN4-deficient mice reported here are on a mixed 129 SvEv×C57BL/6 background whereas the PTPN4-deficient mice that were reported to exhibit motor learning defects are on a 129SvJ background [33]. In both types of PTPN4 mutant, although the precise nature of the genetic mutation differs, PTPN4 gene expression was shown to be extinguished.

In summary, we have thus far been unable to observe any phenotypic abnormalities in PTPN4-deficient, PTPN3-deficient or PTPN4/PTPN3 double-deficient mice either within or without the immune system. Thus, a physiological function for these PTP, that can be consistently demonstrated, awaits discovery. In considering potential other roles, it is of interest that human papillomaviruses specifically target FERM and PDZ domain-containing PTP for degradation during infection and transformation [36]–[38]. One possible explanation for this finding is that FERM and PDZ domain-containing PTP play a role in defense against this type of virus. Notwithstanding the fact that human papillomaviruses do not infect murine cells, discovery of the function of these PTP in papillomavirus infection could provide important clues as to their role in normal cellular physiology.
